# Recurrent quantum embedding neural network and its application in vulnerability detection

**DOI:** 10.1038/s41598-024-63021-y

**Published:** 2024-06-13

**Authors:** Zhihui Song, Xin Zhou, Jinchen Xu, Xiaodong Ding, Zheng Shan

**Affiliations:** 1 Information Engineering University, Zhengzhou, 450001 China; 2 Songshan Laboratory, Zhengzhou, 450001 China

**Keywords:** Computer science, Quantum information

## Abstract

In recent years, deep learning has been widely used in vulnerability detection with remarkable results. These studies often apply natural language processing (NLP) technologies due to the natural similarity between code and language. Since NLP usually consumes a lot of computing resources, its combination with quantum computing is becoming a valuable research direction. In this paper, we present a Recurrent Quantum Embedding Neural Network (RQENN) for vulnerability detection. It aims to reduce the memory consumption of classical models for vulnerability detection tasks and improve the performance of quantum natural language processing (QNLP) methods. We show that the performance of RQENN achieves the above goals. Compared with the classic model, the space complexity of each stage of its execution is exponentially reduced, and the number of parameters used and the number of bits consumed are significantly reduced. Compared with other QNLP methods, RQENN uses fewer qubit resources and achieves a 15.7% higher accuracy in vulnerability detection.

## Introduction

There have been many studies using NLP technology to deal with programming languages^1–3^. These methods have been applied to the field of cyber security^4–7^ advancing the development of automated systems, including vulnerability detection systems based on deep learning^8–11^. The continuous development of NLP technology has led to significant improvements in these applications, but also to a massive increase in model complexity (e.g., the number of parameters in GPT models has reached the order of hundreds of billions^12,13^). Training such a model requires huge memory resources and time costs, which has become one of the bottlenecks in classical NLP technology. Such problems also plague applications such as vulnerability detection, as strong performance often means huge costs for complex models with extensive training. Therefore, one desire to find a more efficient computing method to optimize models and reduce costs^14^.

Quantum computing is a computing method with great potential. In quantum computing, qubits are able to represent a superposition of exponentially multiple states simultaneously and allow simultaneous operations on the superposition states. Therefore, it has more powerful information storage capacities and allows performing computations with less computational complexity compared to classical computing^15–17^. So far, there have been many studies on quantum neural networks (QNN). The so-called quantum neural network is a neural network model based on quantum computing that learns by executing a circuit composed of quantum unitary gates containing trainable parameters and optimizing the parameters^18–20^. It inherits the properties of quantum computing, including superposition, interference and entanglement of information carried by qubits. QNN is expected to take advantage of quantum computing to improve the performance of neural networks and reduce costs. This is because it can generate inter-variable correlations that cannot be represented by classical computing, achieve significantly higher effective dimensions and fit data faster on exponentially higher feature spaces^21–23^. Therefore, quantum neural networks are expected to solve the above problems.

However, it is difficult to combine QNN with NLP technology and apply it to vulnerability detection. Because simple combinatorial approaches of text embedding and context-dependent learning migrated from classical NLP techniques do not work in QNN. For example, QRNN^24^ can learn sequential data (such as sequences of digits), but it is unable to handle natural language. To address this difficulty, the Categorical Distributional Compositional (DisCoCat) model^25^ for natural language has been applied to QNN^26^, which has become the common theoretical framework for almost all QNLP methods^27^ However, such methods consume a large amount of qubit resources and their performance on specific tasks still needs to be improved. Therefore, in this paper, we aim to construct a QNN model for vulnerability detection that (a) consumes significantly less memory than classical neural networks and (b) perform better and consume fewer quantum resources compared to existing QNLP methods.

To accomplish this goal, in this work, we propose a trainable encoding method based on parameterized binary index. Using this method, each token of the code sequence is transformed into a small segment of trainable quantum embedding circuit to obtain an effective quantum word embedding. This quantum embedding circuit is used to form a recurrent cell in combination with the quantum weight circuit. And it is successively applied to the iterative inputs of the network to capture the contextual dependencies in the code. On this basis, we construct a Recurrent Quantum Embedding Neural Network (RQENN) model for vulnerability detection.

Simulation results and analysis on the vulnerability detection tasks show that compared with the classic model, the space complexity of each stage of RQENN execution is exponentially reduced, the number of parameters used is only 0.21% of the classic RNN, and the number of bits consumed is also significantly reduced. Compared with other QNLP methods, RQENN uses fewer qubit resources, runs fewer quantum circuits, performs fewer measurement operations, and achieves 15.7% higher accuracy in vulnerability detection. The results obtained are the state-of-the-art classification performance among reported QNLP methods.

In general, the contributions of this work are as follows:

1. We apply QNN to vulnerability detection, expanding the new way of combining quantum computing with cyber security.
2. We propose a trainable encoding method based on parameterized binary index, which can effectively extract quantum word embeddings.
3. We propose the RQENN model that can be used to process textual data, which opens up a new direction in QNLP technology different from the DisCoCat diagram model.
4. RQENN reduces the memory consumption of classical models; it consumes fewer qubit resources and has higher accuracy than other QNLP methods.

This paper is organized as follows. In the “Background” section, we introduce the vulnerability detection and QNLP technology. In the “Methods” section, we show the trainable encoding method, the RQENN cell, the implementation of the RQENN classification model, and the task flow of vulnerability detection. In the “Results” section, we show simulation results and analysis on vulnerability detection task using RQENN. Finally, in the “Discussion” section, we summarize the paper and look forward to further work.

## Background

### Vulnerability detection

Software vulnerabilities pose a serious threat to network security. Traditional vulnerability detection methods often rely on static analysis (e.g., vulnerability rules, symbolic execution) or dynamic analysis (e.g., fuzzing test, taint analysis) techniques. However, these methods have certain limitations^28^. Static analysis methods are often limited by the complexity of the code and have many false positives and false negatives. Dynamic analysis methods consume a lot of time due to the running of the program and are sensitive to test data. Therefore, they are difficult to meet the current complex and changing software security requirements.

In recent years, the excellent performance of deep learning on NLP tasks has inspired researchers to use neural networks to build automated vulnerability detection systems and achieved remarkable results. Vulnerability detection methods based on neural networks aim to extract high-dimensional features of the code through neural networks to make a judgment on the existence of vulnerabilities at the level of code slices, functions, or statements. These studies extract effective abstractions from raw code data by processing code as text^29,30^, extracting vulnerability function/API-related code slices (e.g., code gadgets)^31–33^, and extracting graph structural information such as PDG and AST of the code^11,34–36^ and other methods. They are converted into vectors for input to the neural network by further preprocessing. Similar to traditional NLP tasks, vulnerability detection also requires neural networks to be able to memorize sequential and semantic information about the code, because the context information of the code often contains the conditions for triggering the vulnerability. As a result, CNNs, RNNs, and some GNNs are frequently used^28,37,38^, and some large language models^1,39,40^ migrated to code also come into play.

As a clear example, ref.^30^ implements the first deep learning-based vulnerability detection system, proposing a code intermediate representation "code gadget". A code gadget is a collection of code statements that are semantically related to some manually defined vulnerability characteristics in a program (e.g., an API call). The code gadget extracted from source code is regarded as text, and each token is encoded into a word vector through word embedding then input into BLSTM network to capture the sequence information of the code. Eventually the trained model will make a detection of whether the code contains vulnerabilities.

### QNLP technology

In recent years, the size of classical NLP models has been increasing, with neural networks reaching even hundreds of billions of parameters^12,13^. The strong performance of classical NLP models often means complex models and huge memory consumption, and there seems to be an irreconcilable contradiction between model performance and memory consumption, which hinders their further application in vulnerability detection. The reason for the huge memory consumption of these models is that these models suffer from dimensionality catastrophe when dealing with high-dimensional, complex data. When the dimensionality of the data increases, the required computational resources and storage space increase dramatically. At the same time, a large number of parameters of the model are involved in the forward propagation, and the calculated hidden layer variables continue to accumulate, resulting in rapid memory consumption.

In order to reduce the computing cost of current NLP methods, some studies combine QNN with NLP to take advantage of the huge benefits of quantum computing in information storage and parallel acceleration, which is called quantum natural language processing (QNLP)^26,41–43^. Reference^26^ encodes words and phrases into quantum states and processes based on the DisCoCat model and implements it using variational quantum circuits (VQC). The core of the approach is to consider DisCoCat as a tensor network model of natural language meanings, which can be represented as string diagrams and further transformed into quantum circuits. This method has been proven to have certain advantages in theory^25,41^, so the DisCoCat model became a common theoretical framework for almost all QNLP methods. But it has only been tested on very small datasets^43–45^ and has not yet been applied to real-world tasks. In addition, some QNNs for processing sequence data have been proposed, such as RQNN^24^. However, they cannot be useful for specific tasks related to text or language, because QNN is unable to obtain quantum word embeddings from textual information as efficiently as classical models using a combination of one-hot and word embedding methods. There are also methods based on the classical network structure (e.g., QLSTM^45–47^), which perform NLP tasks by replacing weight parameters with VQCs, and this hybrid quantum-classical network structure is also considered as a QNLP model in a broader sense. The proposed methods have promoted the development of QNLP, but they are still far from practical application. The results on small tests^27^ show that the exploration of QNLP technology is still full of challenges.

## Methods

In this section, we first introduce the composition and principles of the important components of RQENN including the trainable encoding method based on parameterized binary index and recurrent cell. Then we introduce the RQENN-based classification model. Finally, we present the task flow of applying RQENN classification model to vulnerability detection.

### Trainable encoding method

**Figure 1:**
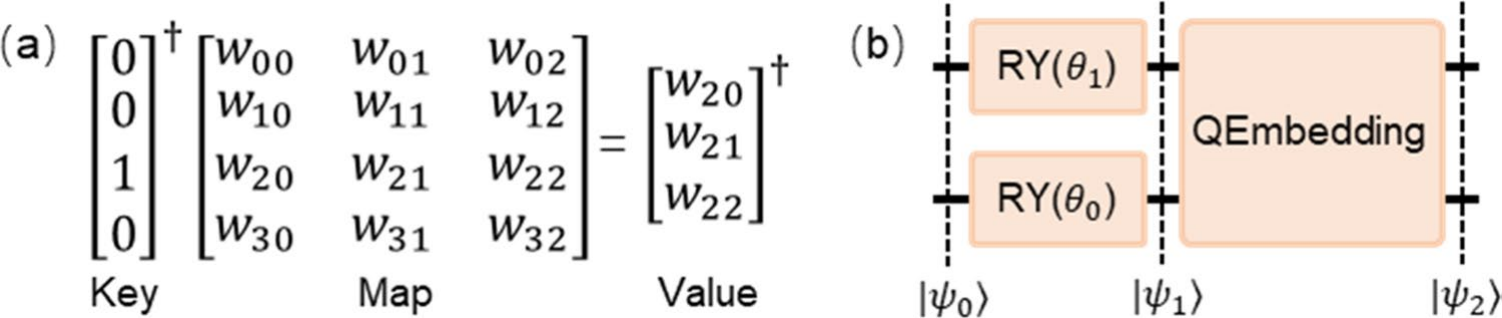
(**a**) The process of token 'NULL' being encoded into a quantum circuit. The $|{\psi }_{1}\rangle$ obtained after rotation input layer is treated as a quantum one-hot vector. It further applies QEmbedding to obtain $|{\psi }_{2}\rangle$ which can be treated as a quantum dense vector used as token representation. (**b**) Classical word embedding computation process. The one-hot vector is treated as a key to query value in the map of weights.

Classical neural network models for processing NLP tasks first need to tokenize the text and build a word dictionary, according to which the text is converted into a digital index sequence of words. Each digital index corresponds to a one-hot vector, which are transformed into dense vectors by word embedding methods involved in the network training to obtain a more accurate vector representation. However, similar methods migrated to QNN do not work. Specifically, in the classical model, the one-hot vectors are sparse and orthogonal, which means that when word embedding is performed, each word gets only some of the weights from the embedding weight matrix $W$ as the representation vector. This process can be viewed as using the one-hot vector as the key to query the corresponding value in the weight map $W$, as shown in Fig. 1a. Thus, in the case of random initialization of weights, the initial representation vectors of all words are uncorrelated, and they establish lexical connections as the training process proceeds. However, in the quantum model, due to the properties of quantum superposition and entanglement, the quantum state obtained from encoded words (e.g., $|{\psi }_{1}\rangle$ in Fig. 1b) is difficult to be sparse and orthogonal as the classical one-hot vectors. This implies that the initially encoded quantum state of each word has some kind of connection, and the use of this quantum state as the "key" inevitably leads to the "value" obtained from the query being related to all the elements in the unitary matrix, which contains various non-semantic connections. The use of this quantum state as the "key" inevitably leads to a query that yields a "value" that is related to all the elements of the Missy's orthogonal weight matrix, and the results obtained contain various non-semantic connections. This prevents QNN from learning the semantics of words through the quantum embedding method.

To address this problem, we propose a trainable encoding method based on parameterized binary index to encode code tokens as quantum state data and efficiently learn the semantics of the tokens. The specific steps are as follows:

Step I: Tokenize source code into tokens to create a dictionary and then tokens are mapped to numeric indexes.

Step II: Convert the numeric indexes from decimal to binary representation. For a dictionary containing $N$ words, an index is represented by an $n=\lceil lo{g}_{2}N\rceil$ bits binary numbers.

Step III: Replace "0" and "1" in the binary number indexes with the trainable parameters "${\theta }_{0}$" and "${\theta }_{1}$", forming parameterized binary indexes.

Step IV: Encode parameterized binary index using $n=\lceil lo{g}_{2}N\rceil$ qubits. Each bit of the input is encoded to the corresponding qubit through an Ry rotation gate.

Step V: Add a trainable layer containing parameters to the quantum circuit as a quantum embedding (QEmbedding) implementation.

The Ry rotation input layer and the QEmbedding layer in the above steps together form the trainable encoding layer.

As a simple example, for the following source code training set:

$$\left[ {\text{``}{\text{VAR1 }} = {\text{ NULL''}}, \, \text{``}{\text{VAR2 }} = {\text{ NULL''}}, \, \text{``}{\text{VAR1 }} = {\text{ VAR2''}}} \right],$$

we can build such a dictionary to map all code tokens to numeric indexes:

$$\{ \text{`}= \text{'} : \, 0,\text{`}{\text{VAR1'}} :{ 1},\text{`}{\text{NULL'}} :{ 2},\text{`}{\text{VAR2'}} :{ 3}\} .$$

These numeric indexes are further converted to parameterized binary indexes:

$$\{ \text{`}= \text{'} : \, {\theta }_{0}{\theta }_{0},\text{`}{\text{VAR1'}} :{ {\theta }_{0}{\theta }_{1}},\text{`}{\text{NULL'}} :{{\theta }_{1}{\theta }_{0}},\text{`}{\text{VAR2'}} :{ {\theta }_{1}{\theta }_{1}}\} .$$

Next, we determine the angles of the Ry gates and construct the quantum circuit based on the parameterized binary index of the word to be encoded. Taking encoding token 'NULL' as an example, as shown in Fig. 1b, its corresponding indexes ${\theta }_{1}{\theta }_{0}$ are encoded bit-by-bit to a circuit with 2 qubits as the Ry gates’ angles. Then a QEmbedding layer is further added to jointly form the quantum trainable encoding circuits.

As Eqs. (1–3) shown below. $|{\psi }_{0}\rangle$ is the initial state. The quantum circuit encodes the index ${\theta }_{1}{\theta }_{0}$ into the quantum state $|{\psi }_{1}\rangle$ by rotation input layer. It is a ${2}^{n}$-dimensional vector. It has $N$ different cases, corresponding to $N$ possible combinations of the input rotation layer parameters. The parameters "${\theta }_{0}$" and "${\theta }_{1}$" are involved in the training process of QNN to eliminate possible inherent connections, so that $|{\psi }_{1}\rangle$ has the same function as the classic one-hot vector. The one-hot vector is a form transformed by symbols that is easy to use by the classic network model, and the obtained $|{\psi }_{1}\rangle$ is a form transformed by symbols that is easy to use by QNN. This is the unique aspect of trainable encoding based on parameterized binary index and the key to improving model performance. Next, $|{\psi }_{1}\rangle$ learns lexical connections between encoded words through a trainable QEmbedding layer ${U}_{qe}({{\varvec{\theta}}}_{qe})$, which is similar to the classic word embedding principle. The obtained quantum state $|{\psi }_{2}\rangle$ is described in Eq. (3), where $${U}_{qe}({{\varvec{\theta}}}_{qe})={[\begin{array}{cccc}{{\varvec{u}}}_{0}^{\dagger}& {{\varvec{u}}}_{1}^{\dagger}& {{\varvec{u}}}_{2}^{\dagger}& {{\varvec{u}}}_{3}^{\dagger}\end{array}]}^{\dagger}$$. At this point, the ${2}^{n}$-dimensional dense vectors corresponding to $|{\psi }_{2}\rangle$ are the representations of the words, except that the words are converted from indexes to quantum-friendly quantum state representations instead of classical vector representations.

1 $$|{\psi }_{0}\rangle ={[\begin{array}{cccc}{\varepsilon }_{0}& {\varepsilon }_{1}& {\varepsilon }_{2}& {\varepsilon }_{3}\end{array}]}^{\dagger}$$

2 $$\left|{\psi }_{1}\right.\rangle =\left\{\begin{array}{c}Ry\left({\theta }_{0}\right)\otimes Ry\left({\theta }_{0}\right)\left|{\psi }_{0}\right.\rangle ,\,\, index={\theta }_{0}{\theta }_{0}\\ Ry\left({\theta }_{0}\right)\otimes Ry\left({\theta }_{1}\right)\left|{\psi }_{0}\right.\rangle ,\,\, index={\theta }_{0}{\theta }_{1}\\ Ry\left({\theta }_{1}\right)\otimes Ry\left({\theta }_{0}\right)\left|{\psi }_{0}\right.\rangle ,\,\, index={\theta }_{1}{\theta }_{0}\\ Ry\left({\theta }_{1}\right)\otimes Ry\left({\theta }_{1}\right)\left|{\psi }_{0}\right.\rangle , \,\, index={\theta }_{1}{\theta }_{1}\end{array}\right.$$

3 $$|{\psi }_{2}\rangle ={U}_{qe}({{\varvec{\theta}}}_{qe})|{\psi }_{1}\rangle ={[\begin{array}{cccc}{{\varvec{u}}}_{0}^{\dagger}|{\psi }_{1}\rangle & {{\varvec{u}}}_{1}^{\dagger}|{\psi }_{1}\rangle & {{\varvec{u}}}_{2}^{\dagger}|{\psi }_{1}\rangle & {{\varvec{u}}}_{3}^{\dagger}|{\psi }_{1}\rangle \end{array}]}^{\dagger}$$

Compared with the trainable encoding method based on parameterized binary index, if the binary index obtained in Step II is used for encoding, the fixed angle of the rotation gate (0 or 1) will result in constant non-lexical connections between $|{\psi }_{1}\rangle$ of different words. These connections are brought into training process of the quantum word embedding layer, possibly affecting the normal learning of lexical connections of the code tokens. In fact, it can also make the $N$ quantum states $|{\psi }_{1}\rangle$ orthogonal to each other like classical one-hot vectors by choosing a suitable fixed angle of the rotation gate, this method is called the "orthogonal method". It determines the specific angles "${\theta }_{0}$" and "${\theta }_{1}$" to be used for replacing the binary "0" and "1" before training. By respectively applying $N$ different rotation layers with angles "${\theta }_{0}$" and "${\theta }_{1}$" on $N$ independent quantum circuits, we can obtain $N$ quantum states. We use the gradient descent algorithm to minimize the sum of the absolute values of the two-by-two inner products of these $N$ quantum states under random initialization of the quantum initial states. This approach references the property of mutual orthogonality between one-hot vectors, which ultimately yields ${\theta }_{0}=-\frac{\pi }{2}$ and ${\theta }_{1}=\frac{\pi }{2}$, and encoding using this value will make $|{\psi }_{1}\rangle$ as orthogonal as possible for different tokens. But there are also differences between QEmbedding and classical embedding. Each element of the weight matrix in classical embedding is a trainable parameter, while QEmbedding only controls the changes of the matrix through a small number of parameter-containing unitary gates. It cannot be proved that the always orthogonal $|{\psi }_{1}\rangle$ is more helpful for learning $|{\psi }_{2}\rangle$. Therefore, in this paper, we add "${\theta }_{0}$" and "${\theta }_{1}$" as trainable parameters to the learning process of RQENN, which is the reason for using parameterized binary indexes in Step III. We will show in the Results section the performance of the model when using trainable encoding based on binary index, orthogonality method, and parameterized binary index as data inputs, further demonstrating the effectiveness of the proposed methods.

### RQENN cell

The trainable encoding method defined in the above section is a crucial component in the construction of our recurrent quantum embedding neural network cell. Much like classical RNNs, we define such a cell that will be successively applied to the input presented to the network for capturing contextual connections in the code. More specifically, the cell is comprised of a trainable encoding stage and a working stage, which are used to learn the semantics of input tokens and memorize contextual dependencies, respectively. This cell is applied iteratively in RQENN, and its internal state is passed on to the next iteration of the network. RQENN cells at all time steps share the same trainable parameters.

**Figure 2:**
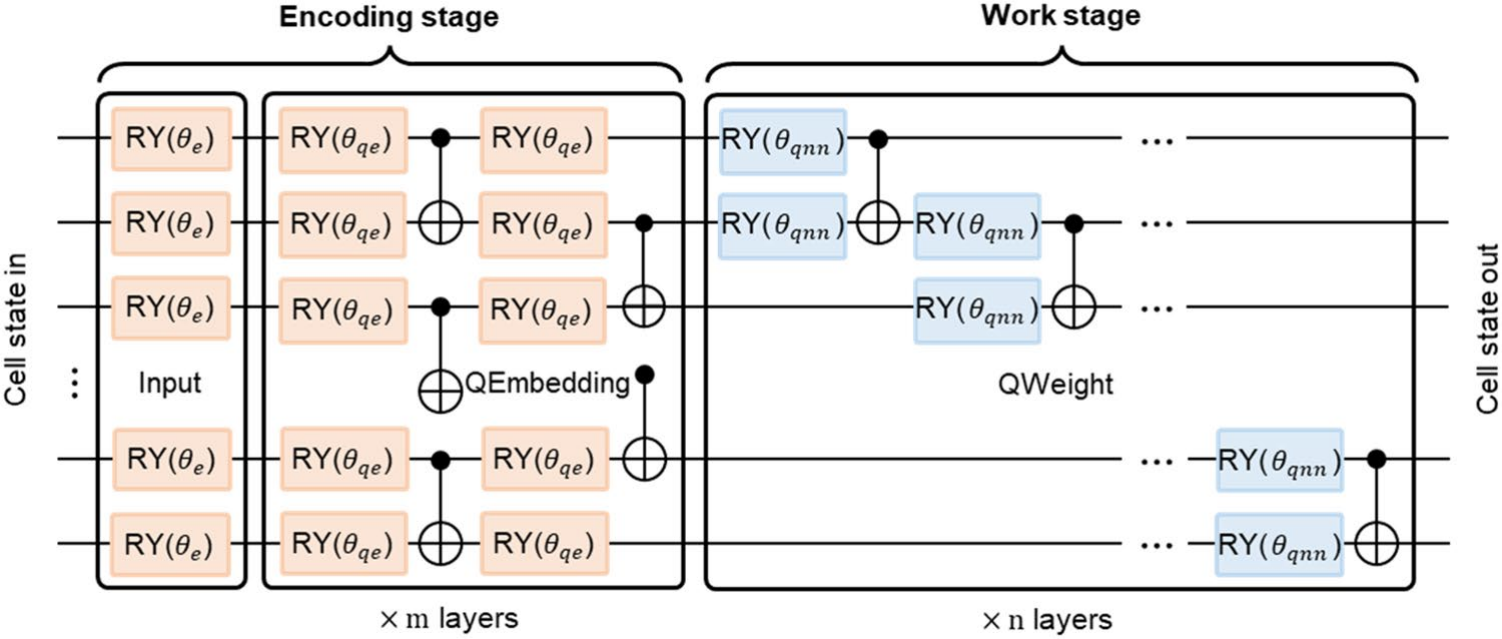
Recurrent quantum embedding neural network cell. It consists of a trainable encoding stage and a QNN work stage, where the principle of the encoding stage is as described in the above section. It transforms the internal state in into the state out at each time step and iterates this process.

Figure 2 illustrates the RQENN cell, which learns the quantum word embedding of the current time step input ${{\varvec{x}}}_{t}=({x}_{{t}_{0}},...,{x}_{{t}_{n}})$ in the encoding stage and combines it with the cell input hidden state $|{\psi }_{t-1}\rangle$ in the work stage to learn the mapping relation from this combined state to the cell output hidden state $|{\psi }_{t}\rangle$. The equation for this process is as follow:

4 $$|{\psi }_{t}\rangle ={U}_{qnn}{U}_{qe}{U}_{in}({{\varvec{x}}}_{t})|{\psi }_{t-1}\rangle$$

where ${U}_{in}$, ${U}_{qe}$ and ${U}_{qnn}$ denote the unitary matrix of the rotation input layer, QEmbedding layer and quantum weight (QWeight) layer, respectively.

The encoding stage uses the trainable encoding method described above. In the rotation input layer, an Ry gate is applied on the ith qubit to rotate the angle to the ith value of the parameterized binary index. In the QEmbedding layer, a $m$ layer ansatz composed of alternate rotation layer and entanglement layer is used to learn the quantum word embedding representation. Each layer of the ansatz consists of 2 rotation layers and 2 entanglement layers consisting of staggered entanglements between adjacent qubits. In the working stage, a $n$ layer one-dimensional alternating layered Hardware Efficient Ansatz^48,49^ was used to build the QWeight layer. This ansatz is implemented by sequentially applying a two-qubit unitary to adjacent qubits. Each two-qubit unitary entangles the last qubit obtained from a previous unitary with the next one. The unique recurrent circuit cell architecture with scalable layer in multi-stage is the key to improving model performance. This two-qubit unitary consists of two Ry gates and a Cnot gate that have been proven effective^50^, and its unitary transformation is described by Eq. (5). We show below the specific implementations of the different network layers by equations. Equation (6) shows the unitary transformation of the rotation input layer, where $t\in \{1,...,T\}$ represents the time step. A token is input into the network at each time step, and $T$ is set as the total code length. Equations (7, 8) and Eq. (9) are the unitary transformations of the QEmbedding layer and QWeight layer respectively.

5 $${U}_{l,i}^{\left[2\right]}\left({{\varvec{\theta}}}_{l,i}\right)=Cno{t}_{i,i+1}{\otimes }_{j=0}^{1}R{y}_{i+j}\left({\theta }_{l,i,j}\right), l\in \left\{\text{0,1}\right\} and i\in \left\{0,\dots ,n-2\right\}$$

6 $${U}_{in}\left({{\varvec{x}}}_{t}\right)={\otimes }_{i=0}^{n}\left(R{y}_{i}\left({x}_{{t}_{i}}\right)\right), {x}_{{t}_{i}}\in \left\{{\theta }_{0},{\theta }_{1}\right\} and t\in \{1,...,T\}$$

7 $${U}_{q{e}_{l}}({{\varvec{\theta}}}_{q{e}_{l}})=\prod_{i=1}^{\lfloor (n-1)/2\rfloor }Cno{t}_{2i-\text{1,2}i}{\otimes }_{i=0}^{n-1}R{y}_{i}({\theta }_{l,n+i})\prod_{i=1}^{\lfloor n/2\rfloor }Cno{t}_{2i-\text{2,2}i-1}{\otimes }_{i=0}^{n-1}R{y}_{i}({\theta }_{l,i})$$

8 $${U}_{qe}\left({{\varvec{\theta}}}_{qe}\right)={U}_{q{e}_{1}}\left({{\varvec{\theta}}}_{q{e}_{1}}\right){U}_{q{e}_{0}}\left({{\varvec{\theta}}}_{q{e}_{0}}\right)$$

9 $${U}_{qnn}\left({{\varvec{\theta}}}_{qnn}\right)={U}_{1,n-2}^{\left[2\right]}\left({{\varvec{\theta}}}_{1,n-2}\right)\dots {U}_{\text{1,0}}^{\left[2\right]}\left({{\varvec{\theta}}}_{\text{1,0}}\right){U}_{0,n-2}^{\left[2\right]}\left({{\varvec{\theta}}}_{0,n-2}\right)\dots {U}_{\text{0,0}}^{\left[2\right]}\left({{\varvec{\theta}}}_{\text{0,0}}\right)$$

### Classification model

**Figure 3:**
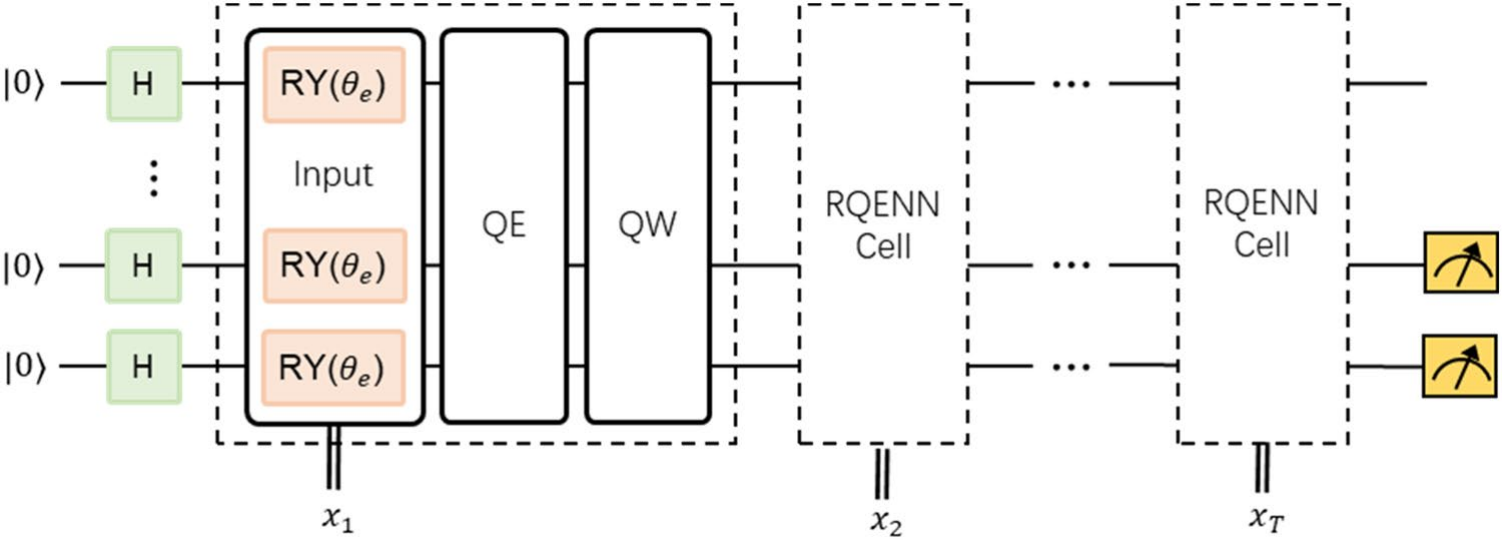
RQENN classifier. The model is built by iteratively applying the same RQENN cell to the input code token sequence. Measurements are performed on the last two qubits separately to obtain the expectation values as classification logits.

We use RQENN cell to build classifiers applied to vulnerability detection. Similar to RNN, RQENN initializes the hidden state at $t=0$ by adding a layer of Hadamard gates initially, and then the RQENN cell is iteratively applied to a sequence of the input source code ${{\varvec{x}}}_{1},{{\varvec{x}}}_{2},...,{{\varvec{x}}}_{T}$ as shown in Fig. 3 to capture the contextual connections in the source code. The entire model also includes measuring the expectation value of a single qubit for the last two qubits. This expectation value is described as Eq. (10):

10 $${E}_{i}\left({\varvec{X}},{\varvec{\Theta}}\right)=\langle {0}^{\otimes n}|{H}^{\dagger\otimes n}{U}_{QC}^{\dagger}\left({\varvec{X}},{\varvec{\Theta}}\right){\widehat{M}}_{i}{U}_{QC}({\varvec{X}},{\varvec{\Theta}}){H}^{\otimes n}|{0}^{\otimes n}\rangle ,\hspace{0.5em}\hspace{0.5em}i\in \{n-1,n-2\}$$

where ${U}_{QC}({\varvec{X}},{\varvec{\Theta}})={U}_{cell}({{\varvec{x}}}_{1},{\varvec{\Theta}})...{U}_{cell}({{\varvec{x}}}_{T},{\varvec{\Theta}})$ is the a quantum circuit composed of all cells, and ${U}_{cell}({{\varvec{x}}}_{t},{\varvec{\Theta}})={U}_{qnn}({{\varvec{\theta}}}_{qnn}){U}_{qe}({{\varvec{\theta}}}_{qe}){U}_{in}({{\varvec{x}}}_{t},{{\varvec{\theta}}}_{in})$. ${\varvec{\Theta}}$ is the parameter set of the cell, and ${\varvec{X}}=[{{{\varvec{x}}}_{1},\dots ,{\varvec{x}}}_{T}]$ is the input index sequence. ${\widehat{M}}_{i}$ is the operator used to calculate the expectation of the ith qubit, i.e.

11 $${\widehat{M}}_{i}=\left\{\begin{array}{c}I\otimes I\otimes ...\otimes {\sigma }_{z}\otimes I,\hspace{0.5em}i=n-2\\ I\otimes I\otimes ...\otimes I\otimes {\sigma }_{z},\hspace{0.5em}i=n-1\end{array}\right.$$

The two calculated expectation values are used to determine the data category by comparing the numerical magnitudes, and we use them as logits to calculate the cross-entropy loss function for classification.

### Task flow

**Figure 4:**
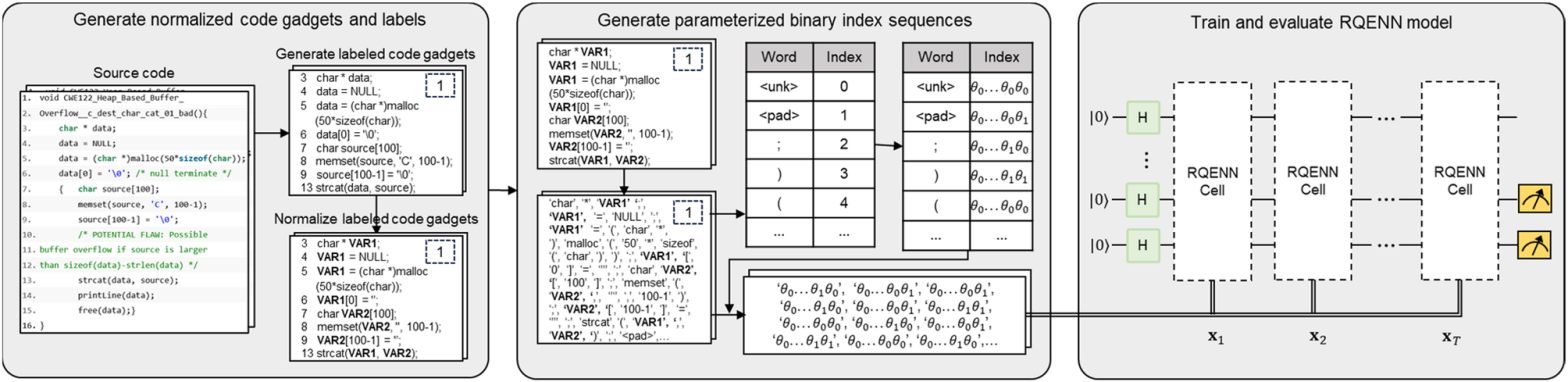
Vulnerability detection task flow. We extract normalized labeled code gadgets from the source code as training data and then generate parameterized binary indexes from them, which are fed into the RQENN classifier. After training, the model can detect the presence of vulnerabilities in the source code.

The goal of our vulnerability detection is to detect whether a program's source code may contain vulnerabilities using the RQENN classifier. In this paper, we perform the vulnerability detection task using the pipeline shown in Fig. 4, which consists of the following three steps:

Step I: Generating normalized code gadgets and labels from source code. First, we extract the data dependency graph (DDG) of the code using the open source code analysis tool Joern. Next, we extract labeled code gadgets based on manually defined vulnerability features. Specifically, we locate the node containing the vulnerable library function/API call in the extracted DDG, such as the "strcat" function shown in left side of Fig.4, and slice the code into small pieces according to the connection to the node. The types of API calls are categorized into forward (e.g., the "recv" function) and backward API calls (e.g., the "strcat" function here) according to whether or not they take external input from a socket, and forward slices and backward slices are generated accordingly. The forward slices obtain the set of statements of the nodes in the DDG that are recursively pointed forward from the API node, and the backward slices obtain the set of statements of the nodes in the DDG that are recursively pointed to the API node. These slices are code gadgets, which are labeled '0' or '1' depending on whether they contain vulnerabilities or not. In the next step we normalized the code gadget. The processing methods include removing comments and strings, normalizing user-defined variable names ('VAR1' etc.) and function names ('FUN1' etc.). Finally, the normalized labeled code gadget is obtained.

Step II: The normalized labeled code gadgets are treated as text data from which parameterized binary index sequences are generated. First, we preprocess the data set, clean the original text, remove punctuation marks and non-ascii characters, etc. Then we split and pad the preprocessed text and build a dictionary, which is converted into a parameterized binary index dictionary according to the method mentioned before. Finally, the token sequences after tokenization are converted into parameterized binary index sequences according to the dictionary.

Step III: Training and evaluating RQENN Models. We input the sequence of parameterized binary indexes into the RQENN model in order, execute the quantum circuit on the simulator or a real machine, and complete the training and validation of the RQENN model according to the quantum circuit learning framework^18^. The model can detect the presence of vulnerabilities in the source code.

## Results

### Dataset description

There is a public dataset we use for vulnerability detection. This dataset is collected from the NVD and SARD vulnerability repositories and provided by Li et al.^31^. It contains C/C++ source codes containing two types of vulnerabilities, buffer overflow and resource management vulnerabilities, as well as source codes that does not contain vulnerabilities. It is used to evaluate the ability of vulnerability detection tools for detecting the presence of vulnerabilities in code. We generated code gadgets from this dataset, and independently selected 1000 data respectively in six intervals ranging from 40 to 100, with the interval length being 10.The number of different categories of data is balanced for each interval. They are used to evaluate the performance of RQENN. Where the average tokens length and maximum length of all 5000 data are 64 and 90 respectively. The number of data with different labels in each interval is balanced. They are used to evaluate the performance of RQENN. Among them, the average token length and maximum length of all 5000 data are 69 and 100 respectively.

### Simulation setup

Our simulations use mindspore as the neural network framework in the Ubuntu environment, and use mindquantum to simulate quantum circuits. All implementations are based on Python language.

We use a fivefold cross-validation method to partition the dataset and test the performance of the model. The size of the dictionary is set to 128, so we use 7 qubits to construct the circuit of RQENN. The time step $T$ is set to the longest length of the token sequence in the selected interval, and the token sequences are padded to the uniform length to support batch processing. We set the training batch size to 64 and the maximum epoch to 10. We use the cross-entropy loss function and the Adam optimizer with a learning rate of 0.01 for training. We use accuracy as the metric to evaluate the model performance. All simulations are performed on a server with an 8-core 3.60 GHz Intel(R) Core(TM) i7-7820X CPU and TITAN RTX GPU.

### Research questions and results

Our simulations are designed to study the following Research Questions (RQ):

RQ1 How does the composition of RQENN affect the model's performance in vulnerability detection tasks?

**Figure 5:**
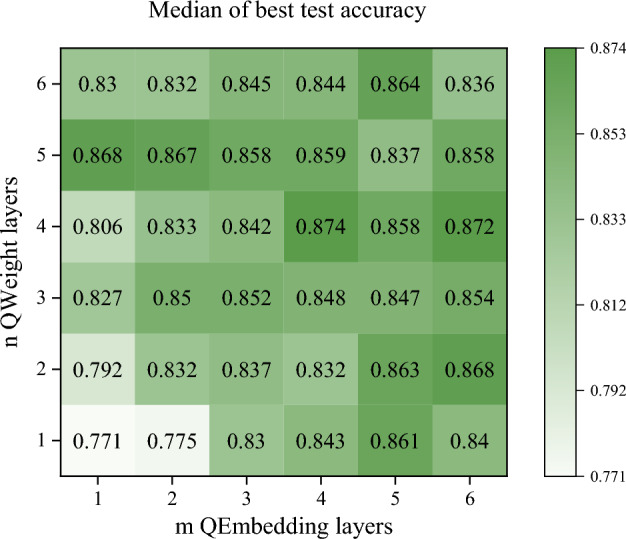
Heatmap of the median of the best test accuracy of the model using $m$ QEmbedding layers and $n$ QWeight layers.

We conducted two ablation experiments on the full dataset using a fivefold cross-validation approach to explore the effect of the composition of RQENN on the model performance, with T = 100 in both simulations. In the first simulation, we conduct an ablation experiment for the number of network layers. We test the effect of using different numbers of QEmbedding and QWeight layers on the performance of the RQENN model in the vulnerability detection task, respectively. Figure 5 shows a heatmap of the median of the best test accuracy for models using $m$ QEmbedding layers and $n$ QWeight layers in the five-fold cross-validation of vulnerability detection task.

The simulation results show that the best median test accuracy of the model tends to increase in general as $m$ and $n$ increase. When $m=4$ and $n=4$, the best median test accuracy of the model reaches the highest 87.4%. In some cases, even if the number of layers increases, the accuracy decreases. For example, the best median accuracy of RQENN with $m=6$ and $n=6$ is lower than that of RQENN with $m=3$ and $n=3$. This suggests that the reason that affects the performance of the model is not only that models with different numbers of layers have different numbers of trainable parameters, but also that different numbers of QEmbedding and QWeight layers themselves have different representational capabilities, which directly affect the size of the solution space of the model, thus leading to different best accuracies.

**Figure 6:**
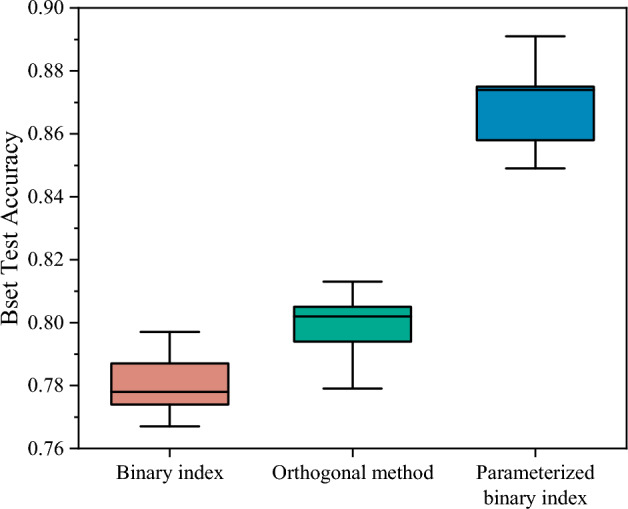
The best test accuracy of RQENN when using trainable encoding method based on binary index, orthogonal method and parameterized binary index.

In the second simulation, we perform ablation experiment for the encoding method. We train the model using original binary index data, data generated by the orthogonality method, and parameterized binary index data inputs, respectively, to study the improvement of RQENN ($m=4$ and $n=4$) by using trainable encoding method. where the orthogonality method fixes the parameters of the parameterized binary index to ${\theta }_{0}=-\frac{\pi }{2}$ and ${\theta }_{1}=\frac{\pi }{2}$. We plot boxplots of the best test accuracy in five-fold cross-validation for models using different data inputs in Fig. 6.

The simulation results show that the median of best test accuracy of the model using the orthogonal method is 77.8%, which is 2.4% higher than the 80.2% of the model using binary index. This shows that using the fixed angles for rotation gates obtained by the orthogonality method (${\theta }_{0}=-\frac{\pi }{2}$ and ${\theta }_{1}=\frac{\pi }{2}$.) instead of the angles (${\theta }_{0}=0$ and ${\theta }_{1}=1$) from the original data reduces the effect of the constant non-lexical connections between $|{\psi }_{1}\rangle$ of different words on the model's ability to learn the meaning of code tokens to some extent. It makes RQENN perform better. And the median of the best test accuracy of the model using parameterized binary index data reached 87.4%, which improved the RQENN by 9.6% compared to the original binary index data. This proves the effectiveness of parameterized binary index. The two angles used in the rotation gates participate in the training process of the model, allowing $|{\psi }_{2}\rangle$ obtained by the quantum word embedding to better represent the meaning of the code.

In summary, the number of QEmbedding and QWeight layers of RQENN and the encoding method affect the performance of the model in vulnerability detection. Within a certain range, the accuracy of the model tends to increase with increasing number of layers and reaches its best at $m=4$ and $n=4$. Using trainable encoding based on parameterized binary indexes is a crucial factor in improving model performance.

RQ2 What are the advantages and limitations of RQENN compared with classical models in vulnerability detection tasks?

In this research problem, we compare the performance of RQENN and the classical RNN model on the vulnerability detection task and analyze the differences in memory consumption between the two in order to explore the advantages and limitations of RQENN over the classical model. In this process, RQENN uses the best performing layers ($m=4$ and $n=4$) from the previous section. As a comparison, the classical RNN network and RQENN perform training with the same settings for all hyperparameters and use the same dictionary. The word embedding dimension and hidden dimension of RNN are both set to 128, which is the same as the vector dimension represented by the quantum state in RQENN.

**Figure 7:**
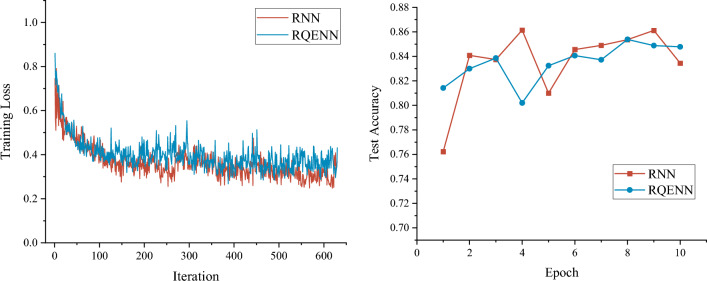
Average training loss and average test accuracy changes of RQENN and RNN during training. We train on the full dataset with a batch size of 64, using the Adam optimizer. We plot the training loss over 630 iterations and the validation accuracy over 10 epochs.

For the performance of the models in the vulnerability detection task, we first conduct simulation on the full dataset, and the results in Fig. 7 show the average training loss and test accuracy changes of RQENN and RNN for 10 epochs of training in a five-fold cross-validation. The simulation results show that the two models demonstrate similar convergence speeds during training, with the RNN having a slightly lower training loss. On the test dataset, RQENN achieves a best test accuracy of 85.4%, which is slightly lower than RNN's 86.1%.

Next, we test two models separately in datasets with different length intervals to investigate the effect of code length variations on model performance. The normalized code gadgets are divided into intervals from length 40 to 100 with interval size $\Delta r=10$. We use dimensionally equivalent RNN as a comparison, and all hyperparameters of the experiment are set the same as RQENN.

**Figure 8:**
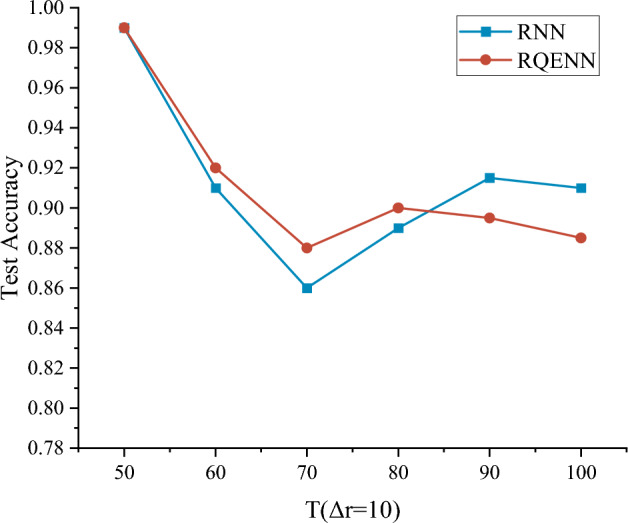
The median of the best test accuracy of RQENN and RNN in different length intervals. Both models are trained for 10 epochs on each interval.

Figure 8 illustrates the median of best test accuracy of both models in different intervals. The results show that as $T$ increases, RQENN outperforms RNN in intervals of $T\le 80(\Delta r=10)$. And in the interval of $T\ge 90(\Delta r=10)$, RNN performed better. This shows that RQENN has better vulnerability detection capabilities on shorter code lengths, and is worse than RNN on longer code. In addition, the test accuracies of RQENN in intervals with smaller size of $\Delta r=10$ are all higher than the test accuracy of $\Delta r=50$ in RQ1, which indicates that data padding also affects the model performance to some extent.

For the differences in memory consumption of the models, due to the fundamentally different computational systems on which they are based, we estimate and compare their memory consumption from multiple perspectives of the equivalent classical information dimension processed and the space complexity at each stage of the model's execution, the number of parameters, and the bit/qubit consumption.

First, we compare the equivalent classical information dimension processed and the space complexity at each stage of the models' execution. As shown in Table 1, the equivalent classical information dimension processed at different execution stages of the two models is the same, but the space complexity is different. At the stage of building dictionary, building a dictionary containing $N$ words requires O($N$) space for RNN, while RQENN requires only O($lo{g}_{2}N$). At the inputting stage, for a code sequence of $T$ tokens where the dimension of each token vector is $N$, the equivalent classical information dimension processed by the models is $N\times T$, the space complexity of RNN is O($N\times T$), while that of RQENN is only O($lo{g}_{2}N\times T$). At the stage of embedding word vectors and getting hidden states, the equivalent classical information dimensions carried by the embedding vectors and hidden states are $d$ and $h$, respectively, and the space complexity of RNN is O($d$) and O($h$), respectively, while the space complexity of RQENN is O($lo{g}_{2}d$) and O $(lo{g}_{2}h)$, respectively. Therefore, the space complexity of RQENN is exponentially reduced compared to the classical model at different stages of the model.

**Table 1:** Classical information amount and space complexity of the two models at each stage of execution.

Model's execution stage	Equivalent classical information dimension	Space complexity of RNN	Space complexity of RQENN
Building dictionary	$N$	O($N$)	O($lo{g}_{2}N$)
Inputting one-hot vectors ($|{\psi }_{1}\rangle$)	$N\times T$	O($N\times T$)	O($lo{g}_{2}N\times T$)
Embedding word vectors ($|{\psi }_{2}\rangle$)	$d$	O($d$)	O($lo{g}_{2}d$)
Getting hidden states	$h$	O($h$)	O $(lo{g}_{2}h)$

Second, we compare the number of parameters of the two models. In classical neural networks, the number of parameters directly determines the memory consumption. The RNN model is set up to have the same word embedding and hidden dimensions as RQENN, but they have a vastly different number of trainable parameters. The weight tensor of h × h dimension in classical RNN can be represented in RQENN using the unitary operator which only contains a small number of trainable parameters. Table 2 demonstrates the number of trainable parameters for both models.

**Table 2:** Comparison of the number of trainable parameters for two models used in vulnerability detection.

Model	RNN	RQENN
Parameters in input layer	0	2
Parameters in word embedding layer	16,384	56
Parameters in the recurrent cell	33,024	48
Parameters in the output layer	258	0
Total number of parameters	49,666	106

The parameters of RNN contain three parts: word embedding, recurrent cell, and output dense layer. It contains a total of 49,666 trainable parameters. The parameters of RQENN contain three parts: input rotation layer, QEmbedding layer, and QWeight layer (recurrent cell), which contain a total of 106 trainable parameters. The number of parameters of the word embedding and recurrent cell used in RNN is 16,384 and 33,024, respectively, while the corresponding number of parameters of the RQENN part is 56 and 48, respectively. Therefore, the number of parameters used in the vulnerability detection model based on RQENN is reduced dramatically compared to the classical model (only 0.21%).

Lastly, we compare the models in terms of the number of classical or quantum bits required. Each parameter of type float 32 in the RNN model occupies 32 classical bits. Thus RNN needs to occupy 1,585,216 classical bits for inference computation. Whereas the RQENN model uses only 7 qubits for inference computation. Although classical bits and qubits are fundamentally different, this comparison also reflects to some extent the huge difference in memory consumption between the two models.

In summary, compared to the classical model, the advantages of the RQENN-based vulnerability detection model are: the RQENN model has a significant advantage in memory consumption, the space complexity at its each execution stage is exponentially reduced, the number of parameters used and the number of bits consumed are substantially less than the classical model. The limitations of the RQENN-based vulnerability detection model are: the RQENN model is slightly less accurate than the classical RNN, and its vulnerability detection performance is more sensitive to the code length compared to RNN, its detection performance on long code is worse than that of RNN.

RQ3 Compared with other QNLP models, does RQENN have more advantages in vulnerability detection tasks?

We conducted extensive simulations on different intervals of the dataset. In addition to the DisCoCat model, we also consider the QLSTM model. Their specific implementation is as follows:

- DisCoCat. We use the lambeq^51^ open source framework to implement DisCoCat diagram for testing. This method adopts a categorical distributional compositional model to construct quantum circuit for language modeling. First, the grammatical reduction of a sentence is interpreted as a diagram that extracts sentence semantics by encoding specific interactions of words according to the grammar. The sentence diagram is then rewritten to simplify the diagram and optimize the use of quantum resources. Finally, depending on the particular parameterization scheme and the specific choice of ansätze, the generated diagrams are converted into specific quantum circuits.
- QLSTM. We implement the QLSTM model proposed in ref. ^47^ and test it. The implementation method is to use variational quantum circuits to replace the trainable parameters in the classic LSTM Cell. Each cell requires 6 VQCs. QLSTM uses classical activation functions. It inputs classical data into VQCs during training, measures the internal state and returns it to classical, and cycles this process between different VQCs. In order to enable QLSTM to perform vulnerability detection tasks, we use a classic embedding layer to obtain the vector representation of words to input into QLSTM, and we add a classic dense layer and a batch normalization layer for post-processing. The word embedding dimension is 128, all VQCs use 7 qubits, and the data is input into VQCs using classical dense layer dimensionality reduction.

We test the detection ability of the three QNLP models on code sets with different length intervals, and the median of the best test accuracy obtained from the five-fold cross-validation are shown in Table 3. Due to the limitation of the simulator’s memory, it is hard to simulate enough qubits for DisCoCat diagrams. The DisCoCat model can only perform classification tasks of T = 20 at most. Therefore, we additionally selected 100 and 250 data in the $T=10(\Delta r=10)$ and $T=20(\Delta r=10)$ data intervals for testing, respectively. The results show that RQENN outperforms DisCoCat and QLSTM in almost all length intervals. All three QNLP models perform well in the detection task in the shortest two length intervals. In the detection task at longer lengths, RQENN shows better accuracy compared to QLSTM. In the task of complete dataset of length interval ranging from 40 to 100, RQENN has a 15.7% higher accuracy than QLSTM, which shows that data padding has less impact on RQENN and the model has better stability. These results show that RQENN is more efficient compared to other existing QNLP methods, and its performance advantage comes from (a) the trainable encoding method based on parameterized binary index substantially enhances the semantic understanding of the model, making it possible to learn code data using a recurrent structure on quantum circuits, and (b) the recurrent structure of RQENN endows the model with a stronger long-term memory capability.

**Table 3:** The median of best test accuracy of three QNLP models on data of different length intervals.

Model	DisCoCat (%)	QLSTM (%)	RQENN (%)
$T=10(\Delta r=10)$	95.0	95.0	100
$T=20(\Delta r=10)$	98.0	90.0	98.0
$T=50(\Delta r=10)$	–	95.0	99.0
$T=60(\Delta r=10)$	–	74.0	92.0
$T=70(\Delta r=10)$	–	68.5	88.0
$T=80(\Delta r=10)$	–	85.5	90.0
$T=90(\Delta r=10)$	–	79.0	89.5
$T=100(\Delta r=10)$	–	76.0	88.5
$T=100(\Delta r=60)$	–	71.7	87.4

Therefore, our proposed RQENN has better performance and stability on the vulnerability detection task compared to other QNLP models. In addition, RQENN uses fewer qubits than DisCoCat. DisCoCat requires an average of 12.8 and 23.4 qubits on the $T=10(\Delta r=10)$ and $T=20(\Delta r=10)$ datasets respectively (varying among sentences), while the number of qubits used by RQENN is only 7. RQENN performs fewer VQCs and measurement operations than QLSTM. One forward propagation of QLSTM requires executing multiple different VQCs, measuring all qubits, and transferring information between classical and quantum, which leads to inefficiency. While RQENN only needs to perform one VQC and measurements on two qubits to complete the task.

## Discussion

In this work, we propose a trainable encoding method based on parameterized binary index. On this basis, we construct a recurrent quantum embedding neural network model for vulnerability detection. Simulations and analysis show that the memory consumption of RQENN is significantly lower compared to the classical model, and RQENN consumes fewer qubit resources and has higher accuracy compared to other QNLP methods. The ablation experiments reveal that using trainable encoding based on parameterized binary index is a crucial factor in improving model performance. Also, the number of QEmbedding and QWeight layers affects the performance of the model in vulnerability detection. These results suggest that RQENN solves to a certain extent the problems of (a) high memory consumption of classical models, (b) difficulty of QNN in handling natural language and (c) poor performance of existing QNLP methods in vulnerability detection. RQENN achieves our preset goals.

However, our work still has some limitations. First, RQENN itself has limitations. This is manifested in (a) RQENN's accuracy in vulnerability detection tasks is slightly lower than that of classical RNN, and this gap is further widened when compared to more advanced methods. For example, the best performance of LSTM in terms of average test accuracy in five-fold cross-validation is 89.7%, and BERT reaches 92.0% (although RQENN still leads in memory consumption). (b) RQENN vulnerability detection performance is more sensitive to code length compared to RNN, with worse detection performance on long code than RNN. Second, RQENN's learning process is based on the Quantum Circuit Learning (QCL) paradigm^18^, which causes it to suffer from similar difficulties as other QNNs employing this paradigm. This is demonstrated by the fact that (a) the QCL learning paradigm is unable to utilize quantum parallelism to process multiple examples at the same time, and thus the training time remains linearly increasing with the amount of data. (b) The performance of QCL-based QNN models is affected by the noise of the quantum hardware. While QNNs are inherently noise tolerant, the effect of noise accumulates as the VQC scale (or quantum volume) increases. Considering the circuit depth of RQENN, the impact of noise is undoubtedly significant, which directly weakens the stability and reliability of vulnerability detection results. (c) Limited by the quantum volume of the quantum hardware, the current QCL-based QNN model has a limitation on the number of qubits and depth of the circuit. RQENN has a deep circuit although it uses a sufficiently small number of qubits. This means that RQENN is only likely to be able to execute small examples on current quantum machines, with limited scalability for larger codebases.

Therefore, RQENN needs to be further improved. First, the trainable encoding method and the structure of RQENN need to be further improved to enhance its semantic comprehension and long-term memory. Second, the circuit depth of RQENN needs to be reduced to increase its scalability. This can be achieved, for example, by encoding multiple consecutive tokens within a single recurrent cell. Finally, to enhance the stability and reliability of the model on real quantum hardware, RQENN needs to be run in quantum hardware with high fidelity to reduce the accumulated noise. A small amount of noise can instead enhance the generalization ability of the model. This approach has realizability for RQENN since it uses only 7 qubits.

In addition, task-related research should be carried out further. First, in the vulnerability detection task, since the real-world has much less vulnerable code than normal one, the impact of dataset imbalance on RQENN needs to be further explored, and strategies such as data augmentation, cost-sensitive learning, sampling and integration learning need to be adopted to ensure the generalization of the results. Second, in order to have a more comprehensive understanding of the performance of RQENN, we need to conduct in-depth studies and detailed analysis of its application in various QNLP tasks on more datasets. We will embark on the above studies in the next step of our work.

In summary, our work expands new approaches of quantum computing for cyber security and natural language processing, and validates new applications of quantum computing in cyber security. We open up a new direction of QNLP technology that is different from the DisCoCat diagram model, and demonstrate the possibility of applying QNLP technology with quantum advantages to real-world tasks. We hope this work can inspire further research on QNLP technologies as well as their real-world applications^41^.

## Data Availability

All the data that support the findings of this study are available from the corresponding authors upon reasonable request.
